# Transition to an In-House Night Float System for Critical Care Fellows: Resident Experience, Morbidity, and Mortality in a Rural Academic Hospital

**DOI:** 10.7759/cureus.17200

**Published:** 2021-08-15

**Authors:** Kyle D Chapman, Varun Badami, Lauren Stawovy, Sana Ali, Mohamad Abdelfattah

**Affiliations:** 1 Medicine/Pulmonary and Critical Care, West Virginia University School of Medicine, Morgantown, USA; 2 Medicine/Pulmonary and Critical Care, Albany Medical Center, Albany, USA; 3 Medicine/Pulmonary and Critical Care, Martin Luther King, Jr. Community Hospital, Los Angeles, USA

**Keywords:** night float, critical care, medical education, rural medicine, academic medicine, resident experience, nocturnal coverage

## Abstract

Background

In-house night call systems for ICUs are frequently implemented to enable hands-on patient care and provide direct supervision of resident physicians at night. Previous studies have highlighted the benefits of an in-house night float (NF) such as minimized time to intervention but failed to consistently demonstrate an improvement in patient outcomes. This study aimed to evaluate the impact of an in-house critical care fellow at night on the resident experience and assess for impact on patient morbidity and mortality.

Methods

An in-house overnight critical care fellow shift was implemented at West Virginia University Hospital in 2018. Resident physicians rotating overnight in the medical ICU (MICU) for six-month periods before and after the intervention were anonymously surveyed. A retrospective chart review of 300 patients admitted overnight to the MICU was performed. Multiple patient outcomes from the pre (2017) and post (2018) intervention periods were collected and compared using a two-sample t-test.

Results

In the post-intervention survey, nearly every element of resident experience improved (availability of support, comfort in performing invasive procedures, and input in treatment plans), and far fewer residents felt overwhelmed relative to the pre-intervention survey. The resident experience markedly improved with the addition of an in-house critical care fellow.

For the retrospective chart review, both groups had similar severity of illness and there was no change in ICU or hospital length of stay. No difference in mortality was found, though the study was underpowered for this outcome. For secondary measures, there was no difference in mechanical ventilation or use days, though more procedures performed were overnight compared to the former staffing model.

Conclusions

Implementation of an in-house overnight critical care fellow shift in the MICU positively impacted resident experience without worsening patient outcomes. The intervention did not worsen measures of morbidity or mortality but did lead to an increased number of procedures performed overnight. The model of in-house NF coverage continues to be preferred by clinicians.

## Introduction

There are different systems utilized in the ICU to enable specialty care for critically ill patients at night. Across the United States, many academic hospitals opt for a model of critical care faculty taking overnight calls in-house, provided sufficient staffing is available. For hospitals with training programs in critical care, fellows often perform the duty of in-house night calls, with faculty backup available from home. Nocturnal intensivist coverage throughout the country appears to be increasing, particularly in academic institutions [1].

The perceived advantages of an in-house physician at night include improvement in the initial resuscitation of the patient, optimization of decision-making, and minimization of delay to medical intervention [2]. The presence of a more experienced in-house physician at night should mitigate uncertainty from cross-coverage issues and enhance the experiential learning of junior residents. Collectively, direct involvement should lead to improved patient outcomes and a better experience for the resident physicians taking night calls. However, a recent systematic review and meta-analysis has shown that in-hospital nocturnal intensivist staffing is not associated with improved ICU mortality or a decreased ICU or hospital length of stay [3].

In contrast to a night float (NF) system of in-house call, the traditional home call option allows critical care specialists to provide supervision of the ICU remotely. This is often a compromise to accommodate limited staffing and the Accreditation Council for Graduate Medical Education (ACGME) work hour limitations. While the physician in this model is on call, he or she may often need to return to the hospital at night for any urgent or emergent patient needs. This model can be demanding for the physician and typically results in interrupted sleep and poor communication between the physician and hospital staff. Moreover, robust evidence demonstrates that sleep loss degrades physician performance and well-being [4].

Despite these outcomes, when compared to intensivists taking overnight calls from home, in-house night calls at a tertiary ICU were preferred by two-thirds of the intensivists queried and they experienced less job and life stress in the latter [5]. The general impression of practitioners of an in-house NF system is that NF tends to be favored. In a neurosurgical residency program that transitioned to an in-house NF system, it was noted that the residents nearly universally had a positive stance towards the NF system, citing improved adherence to duty hours, quality of life, patient outcomes, overall residency training, and fatigue [6].

There is a sparsity of literature that evaluates the ideal night call arrangement for critical care fellows. When looking specifically at pulmonary and critical care medicine fellows and night coverage, the data is virtually non-existent. One study published in 2013 looking at the impact of nocturnal ICU staffing did include trainees in their definition of nighttime intensivist staffing. This was not subdivided to specify the level of training for the trainees (resident or fellow) or to compare outcomes of patients under the care of attending physicians versus physicians in training. Further analysis showed limited benefit from nighttime intensivists for centers that had high-intensity critical care provided by day [7].

In July 2018, our academic institution transitioned from a home call system and to an in-house NF system for critical care fellows covering the medical ICU (MICU). The rationale for this transition was an increasing ICU patient workload, concern for patient safety as the hospital expanded, and alleviating some of the stress of residents taking in-house calls at night. We hypothesized that an in-house NF system for critical care fellows would improve the resident learning experience without compromising patient outcomes when compared to a nocturnal home call system.

## Materials and methods

The study was performed at West Virginia University (WVU) Hospital, a 690-bed rural academic medical center in Morgantown, West Virginia. The two-part study consisted of (1) an anonymous survey of resident physicians who performed overnight MICU shifts, in addition to (2) a retrospective chart review to evaluate the effect of introducing an overnight in-house critical care fellow on outcomes of overnight MICU admissions during the same period.

Implementation of an in-house overnight critical care fellow took place on July 1, 2018, which introduced an NF shift from 1900 to 0700 hours, with the on-call critical care fellow physically present in the ICU, typically for a two-week block with one night off in the middle. The fellow’s primary duty was to supervise overnight MICU admissions performed by residents in addition to assisting with cross coverage and triaging ICU beds for patients from the medical wards, emergency department, and outside hospitals. Prior to the intervention, the critical care fellow took home calls to fulfill these responsibilities. The remainder of the staffing model for ICU coverage was unchanged: two non-intern residents in-house (a mix of internal medicine, family medicine, and emergency medicine) with a specialty trained attending physician on home call.

Approval for the study was obtained by the West Virginia University Office of Research Integrity and Compliance (IRB protocol # 1906608039). Health Insurance Portability and Accountability (HIPAA) waiver of informed consent was approved by the Human Research Protections Program Institutional Review Board for the retrospective chart review as the protected health information of patients was de-identified. For the anonymous resident survey, informed consent was included with the distribution e-mail, emphasizing both the voluntary and anonymous nature of data collection. All procedures performed in the study involving human participants were in accordance with the ethical standards of the institutional research committee and with the 1964 Helsinki declaration and its later amendments.

Resident survey

An email containing a link to an anonymous survey was distributed to the senior residents from West Virginia University who had taken calls in the MICU over the study periods, following their completion of the ICU rotation. A consent was included in the email and explained further that completion of the survey was anonymous and voluntary. After confirming their participation, residents were asked to rate their experience in several aspects of the rotation: adequacy of available supervision, the sensation of being overwhelmed during the night shift, degree of comfort in performing procedures, availability of supervision in performing procedures overnight, availability and helpfulness of the fellow, and the degree of fellow input in the plan of care for patients (see supplemental material). Additionally, residents were encouraged to comment on other methods to improve the ICU overnight resident shift to contribute to any future changes. Surveys of residents rotating between July and December 2017 were compared to surveys of residents rotating between July and December 2018. 

Retrospective chart review

Three hundred patients were included in the study. One of the primary outcomes of the study was ICU length of stay. Based on our institution’s volume and historical data we estimated that a sample size of 300 would provide 80% power to detect a change in 20% ICU length of stay at a two-sided significance level of 0.05. This difference in ICU length of stay was defined as clinically significant based on institutional bottlenecking in bed availability, which has historically varied by multiple hours, as well as previous studies using a rate ratio of 1.2 for the outcome of time to ICU discharge. The study was not powered for mortality, as previous definitive randomized clinical trials and meta-analyses had been inconclusive, and given our ICU’s baseline mortality one model suggested a study population of more than 1000 patients to detect a 5% mortality, an effect size beyond previous quality improvement studies or pilots.

Inclusion criteria were adult patients admitted to the MICU between the hours of 1900 and 0700. Exclusion criteria were any patients admitted to a non-medical intensive care service (surgical or neuro-critical care teams) or admission between 0700 to 1900 hours. The pre-intervention cohort included 150 patients selected from July to December 2017. The post-intervention cohort included 150 patients selected from July to December 2018. The periods were chosen to adjust for seasonal variation in illness severity to ensure cohorts were otherwise comparable. Monthly lists of patients admitted to the MICU were generated and patients were randomized within the lists. From each randomized monthly list, the first 25 patients meeting inclusion criteria were included in the study. The primary variables collected were ICU length of stay, hospital length of stay, and ICU mortality. Secondary variables collected were total days on mechanical ventilation, the number of days requiring vasopressor support, the maximum number of vasopressors at any point in the ICU stay, ICU admission diagnosis, the total number of procedures performed during the overnight shift of admission, and Sequential Organ Failure Assessment (SOFA) scores from time of admission. 

Statistical analyses

Resident survey responses were on a scale of five choices, ranging from 1 - “Strongly Disagree” to 5 - “Strongly Agree”, with a 3 as a neutral “Neither Agree/Disagree” response. Scores per question were averaged and compared between the two cohorts using Likert scale analysis and a 1-sample T-test with a population mean of 3 (neutral response). For the retrospective chart review, a P-value of 0.05 was used to show statistical significance. P-values were calculated using the chi-squared test for categorical variables and Welch’s t-test for continuous variables.

## Results

Resident survey

**Figure 1:**
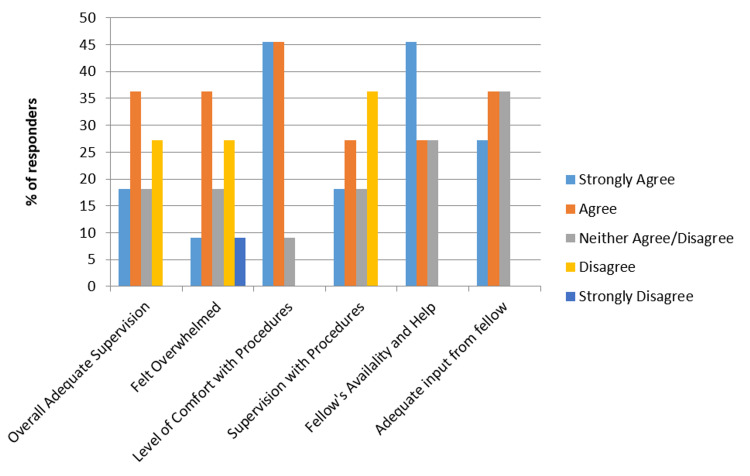
Residents' experience in 2017, prior to implementation of in-house night float fellow support

**Figure 2:**
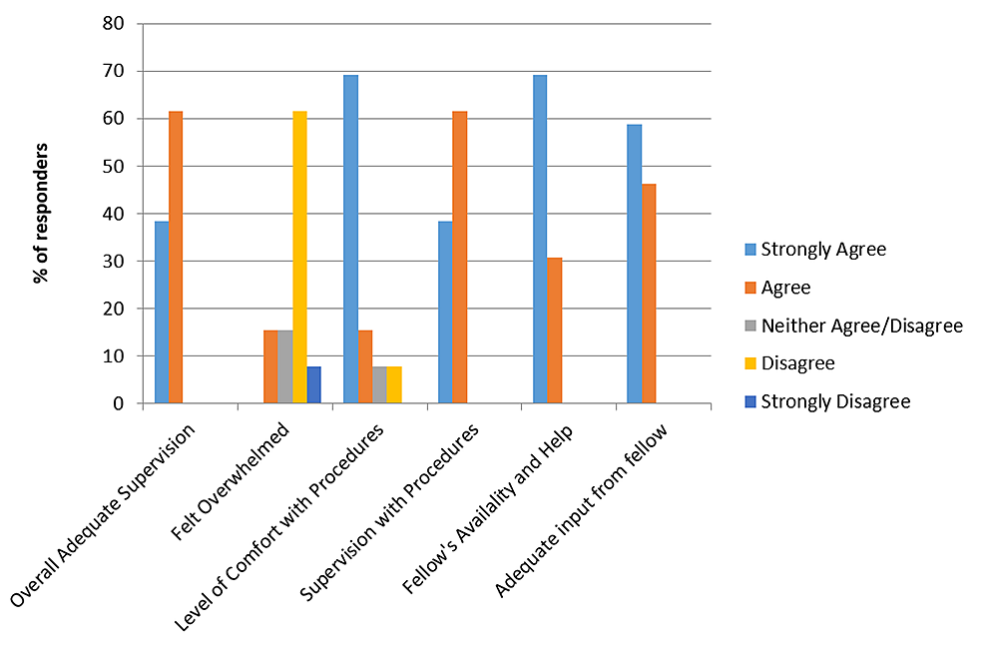
Residents' experience in 2018, following implementation of in-house night float fellow support

Responses were collected from the anonymous survey of senior residents who completed overnight MICU shifts during the July to December 2017 pre-intervention period and the July to December 2018 post-intervention period. We received responses from 12 residents during the pre-intervention period (54.5% response rate) and 13 responses for the post-intervention period (46.4% response rate). Results of the resident surveys for 2017 and 2018 are graphically shown in Figure 1 and Figure 2.

Compared to the cohort working without an in-house critical care fellow, residents with an NF critical care fellow present indicated gains for adequacy of supervision, improved comfort in performing procedures, increased level of supervision while performing procedures, better fellow availability, and increased fellow input on treatment plans. Post-intervention, residents were less likely to feel overwhelmed on their night shift in the ICU.

We analyzed resident responses from the post-intervention period (2018) using a 1-sample t-Test with statistically significant results, as shown in Table 1. Every question on the survey regarding resident experience had a statistically significant change after the addition of an in-house critical care fellow.

**Table 1:** Resident responses following night float intervention in 2018 Likert scale: 1. Strongly Disagree, 2. Disagree, 3. Neither Agree/Disagree, 4. Agree, 5. Strongly Agree Likert scale Analysis using 1-sample t-Test with a population mean of 3 i.e.: Neither Agree/Disagree

Residents response from survey	Mean (SD)	Likert scale	p-Value
Overall satisfaction	4.38 (0.5)	Agree	<0.001
Felt overwhelmed	2.38 (0.86)	Disagree	0.025
Comfort with procedure	4.46 (0.96)	Agree	0.001
Adequate supervision with procedures	4.38 (0.5)	Agree	<0.001
Fellow’s availability and help	4.69 (0.48)	Strongly Agree	<0.001
Adequate input from fellow	4.53 (0.5)	Strongly Agree	<0.001

Retrospective chart review

The overnight admission cohort of 150 patients from July to December 2017 was compared to the overnight admission cohort of 150 patients from July to December 2018. Baseline demographics of the 2017 pre-intervention cohort (before NF) and 2018 post-intervention cohort (after NF) and are shown in Table 2. There was no statistically significant difference between the age, sex, or BMI of the two groups. When the type of admission (direct admission from an outside hospital, admission through the emergency department, or transfer from the floor) between the two groups was compared, no statistically significant difference was noticed (Table 2). There were statistically significant differences in admitting diagnoses, possibly influenced by the presence of the in-house fellow. Prior to the intervention, resident physicians placed admission orders, which included the admitting diagnosis, whereas after the intervention this was more frequently done by the ICU fellow and with greater specificity. Using SOFA score as an approximation of severity of illness, there was no statistically significant difference between the two groups.

**Table 2:** Comparison of baseline characteristics of 2017 cohort and 2018 cohort SOFA = Sequential Organ Failure Assessment; GI = Gastrointestinal

	2017 (before night float)	2018 (after night float)	P-Value
Mean age	60.3 ±16.5	60.3 ±16.9	0.98
Mean BMI	31.5 ±11.8	30.8 ±9.7	0.57
Obesity (BMI >30)	62 (41.3%)	69 (46.0%)	0.42
Female	67 (44.7%)	71 (47.3%)	0.64
Male	83 (55.3%)	79 (52.7%)	0.64
Admission type			
Direct admit to ICU	79 (52.6%)	88 (58.7%)	0.3
Admission from emergency department	32 (21.3%)	24 (16.0%)	0.23
Transfer from another service	39 (26.0%)	38 (25.3%)	0.9
Mean SOFA Score	7.65 ±0.6	6.82 ±0.6	0.064
Admitting Diagnosis			
Sepsis/ Septic Shock	38 (25.3%)	18 (12.0%)	0.003
Hypoxic Respiratory failure	56 (37.3%)	33 (22.0%)	0.004
GI bleed	8 (5.3%)	18 (12.0%)	0.04
Cardiac Arrest	9 (6.0%)	8 (5.3%)	0.80
Drug Overdose	3 (2.0%)	6 (4.0%)	0.31
Other	36 (24.0%)	67 (44.7%)	<0.001

Primary and secondary variable comparisons between the pre and post-intervention cohorts were performed (Table 3). The only statistically significant difference was observed in the total number of procedures performed for new admissions overnight. The average number of procedures in the pre-intervention cohort and post-intervention cohort were 0.186 and 0.380 respectively, (p-value 0.005). Means were calculated for ICU length of stay and hospital length of stay for the pre and post-intervention cohorts demonstrating no statistically significant difference. Mechanical ventilation days, vasopressor duration, the maximum number of vasopressors at a given time, and hospital mortality were without statistically significant differences between pre and post-intervention cohorts. The average ICU length of stay in the pre-intervention cohort and post-intervention cohort was 3.61 (95% CI 1.64 - 4.19) and 3.99 (95% CI 4.63 - 3.63) days respectively, demonstrating no statistically significant difference (p-value of 0.39). Similarly, mean hospital length of stay in the pre-intervention and post-intervention cohorts were 12.85 (95% CI 10.71-14.97) and 12.2 (95% CI 10.05-14.28) respectively, demonstrating no statistically significant difference (p-value of 0.65).

**Table 3:** Effects of in-house night float fellow on length of stay, mechanical ventilation, vasopressors, and number of procedures

	2017 (before night float)	2018 (after night float)	P-Value
Mean ICU length of stay (days)	3.61 (95% CI 1.64 - 4.19)	3.99 (95% CI 3.63-4.63)	0.39
Mean hospital length of stay (days)	12.85 (95% CI 10.71-14.97)	12.2 (95% CI 10.05-14.28)	0.65
Mortality	22 (14.6%)	21 (14%)	0.87
Mean days on mechanical ventilation	2.12 (95% CI 1.51-2.72)	2.00 (95% CI 1.17- 2.84)	0.83
Duration of vasopressor support (days)	0.96 (95% CI 0.68-1.23)	0.74 (95% CI 0.50-0.98)	0.23
Mean number of vasopressors	0.67 (95% CI 0.49-0.85)	0.55 (95% CI 0.38-0.71)	0.31
Number of overnight procedures	0.18 (95% CI 0.10-0.27)	0.38 (95% CI 0.27-0.48)	0.005

Furthermore, the mean number of mechanical ventilation days, vasopressor days, the maximum number of vasopressors at a given time, and in-hospital mortality were calculated demonstrating no statistically significant difference. In the pre-intervention cohort, the mean number of mechanical ventilation days was 2.12 (95% CI 1.51-2.72), the average duration of vasopressors was 0.96 days, and the average maximum number of vasopressors was 0.67. In the post-intervention cohort, the mean number of mechanical ventilation days was 2.00 (95% CI 1.17- 2.84), the average duration of vasopressors was 0.75 days, and the average maximum number of vasopressors was 0.55. The P-values for comparisons ranged from 0.23 to 0.83, demonstrating no statistically significant differences.

## Discussion

The need to provide critical care coverage overnight exists for every ICU in the nation. However, overnight shift work is not without health risks, as it has been designated a 'probable carcinogen' by the World Health Organization [8]. The model of call system for intensivist night coverage varies from hospital to hospital, with many factors to consider. The best model of night call for critical care fellows has not been thoroughly evaluated. To our knowledge, this study is the first to formally assess an in-house NF system for critical fellows and its effect on both resident experience and patient outcomes.

After implementation of an in-house NF system for critical care fellows in July 2018, there was no appreciable change in ICU or hospital length of stay, or duration of mechanical ventilation or vasopressors when compared with a home-call NF system. Our findings are consistent with recent similar data assessing the impact of nocturnal staffing by critical care attending physicians [3]. Of note, our study did show a statistically significant increase in the number of procedures performed overnight with an NF fellow. We suspect that this was likely due to the presence of an experienced critical care fellow who either encouraged or performed more procedures overnight. As noted above, the admitting diagnoses between the two cohorts showed a statistically significant difference in the number of patients admitted with sepsis/septic shock, hypoxic respiratory failure, and GI bleed. The difference between admitting diagnoses in the post-intervention cohort is most likely due to increased specificity of admission diagnosis (see Table 2) with the in-house NF fellow (for example, using an admitting diagnosis of cholangitis instead of septic shock). Though the admitting diagnoses were different, the severity of the illness was comparable. 

The lack of improved patient outcomes should not necessarily be taken as evidence that in-house NF systems are inherently non-beneficial. Traditional home-call night coverage can require multiple interruptions of sleep for practitioners and eventual sleep deprivation, which has been shown to affect cognitive function and is associated with a higher level of stress in physicians [9]. Thus, it is not surprising that surgical residents previously surveyed reported their preference for an in-house NF system and had a very positive perception of this model [10]. Obstetrics and gynecology residents additionally felt that their quality of life was improved with this system in a previous study [11].

Our survey of senior residents continued to display this preference as there was a positive shift in educational experience on ICU nights with the presence of an in-house critical care fellow. Additionally, the residents reported increased comfort when performing procedures overnight and felt less overwhelmed overall during their shift. In total, the whole resident experience with an in-house critical care fellow appeared to have positively improved. These findings are contrary to a previous resident study, in which residents felt that NF systems lead to lost teaching and learning opportunities [12]. The presence of an in-house critical care fellow may also subtract from the residents’ own hands-on experience at night, as they previously had more autonomy in medical decision-making. However, this was not properly assessed in our study. 

Our retrospective analysis does have some limitations. Despite randomization of the monthly data, selection bias was still possible as we enrolled patients sequentially until we met our pre-set 25-patient quota per month, thus all patients admitted each month were not included in our analysis. As noted in the methods, a larger than feasible cohort would have been needed to show a change in mortality. As a limited scale study, finding no statistically significant worsening of ICU length of stay combined with the significant improvement in resident experience is a net positive. More studies are warranted to evaluate any possible mortality benefit of in-house NF coverage for critical care fellows. 

Our survey was potentially affected by recall bias as it was in some cases completed several months after residents had finished their MICU rotation and the combined response rate was around 50%. The limited response rate for both cohorts perhaps selected out those with strong opinions. Our study was also limited to a single-center experience, which may affect its generalizability. We did not specifically address the intervention’s impact on resident burnout, which could have been a compelling narrative. 

For further investigation, evaluation of the fellows’ experience before and after the intervention may provide new insight. Assessment of involved fellows could reveal changes in workflow issues such as communication to subspecialty services, timeliness of appropriate care, learning, and efficiency. A previous study did show that the presence of an in-house critical care fellow in a surgical ICU significantly improved resident-to-fellow communication of key patient cardiorespiratory events at night and led to fewer communication errors [13].

Overall, despite the lack of improvement for specific clinical variables, an in-house NF system for critical care fellows was well received by residents working overnight in the ICU. There may be additional benefits to patients not uncovered by our study, and further investigation is needed.

## Conclusions

The MICU of WVU Hospital transitioned to an in-house overnight call system for critical care fellows in July 2018. When comparing the in-house fellow call system to the previous overnight home call system, the residents reported an improved experience during their overnight shifts in the ICU. We did not find a significant change in ICU or hospital length of stay, ICU mortality, mechanical ventilation days, or vasopressor days. However, more overnight procedures were completed with the NF fellow present. Our findings are consistent with previous studies for other specialties. 

## Appendices

Following verification of participation in overnight calls during either the pre or post-intervention time frame, residents were asked the following questions regarding their overnight ICU call:

1. I felt that I had adequate supervision available to me during my MICU shift.

2. I felt overwhelmed during my MICU night shift.

3. I felt comfortable performing procedures during my MICU night shift.

4. I felt that I had adequate supervision for procedures during my MICU night shift.

5. I felt the backup resident was helpful during my MICU night shift.

6. I felt that the MICU fellow was available and helpful during my MICU night shift.

7. I felt the MICU fellow provided adequate input when developing plans of care for patients during my MICU night shift.

Options choices: Strongly Agree, Agree, Neither Agree/Disagree, Disagree, Strongly Disagree

MICU: Medical intensive care unit
